# A Novel Approach for Predicting Atrial Fibrillation Recurrence After Ablation Using Deep Convolutional Neural Networks by Assessing Left Atrial Curved M-Mode Speckle-Tracking Images

**DOI:** 10.3389/fcvm.2020.605642

**Published:** 2021-01-22

**Authors:** Yi-Ting Hwang, Hui-Ling Lee, Cheng-Hui Lu, Po-Cheng Chang, Hung-Ta Wo, Hao-Tien Liu, Ming-Shien Wen, Fen-Chiung Lin, Chung-Chuan Chou

**Affiliations:** ^1^Department of Statistics, National Taipei University, Taipei, Taiwan; ^2^Department of Anesthesia, Chang Gung Memorial Hospital, Taipei, Taiwan; ^3^Division of Cardiology, Department of Internal Medicine, Chang Gung Memorial Hospital, Linkou, Taiwan; ^4^Chang Gung University College of Medicine, Taoyuan, Taiwan

**Keywords:** atrial fibrillation, deep convolutional neural network, radiofrequency ablation, speckle tracking longitudinal strain, recurrence

## Abstract

**Aims:** Curved M-mode images of global strain (GS) and strain rate (GSR) provide sufficiently detailed spatiotemporal information of deformation mechanics. This study investigated whether a deep convolutional neural network (CNN) could accurately classify these images in patients with atrial fibrillation (AF) who underwent radiofrequency catheter ablation (RFCA) with different outcomes.

**Methods and Results:** We retrospectively evaluated 606 consecutive patients who underwent RFCA for drug-refractory AF. Patients were divided into AF-free (*n* = 443) and AF-recurrent (*n* = 163) groups. Transthoracic echocardiography was performed within 24 h after RFCA. Left atrial curved M-mode speckle-tracking images were acquired from randomly selected 163 patients in AF-free group and 163 patients in AF-recurrent group as the dataset for deep CNN modeling. We used the ReLu activation function and repeatedly performed CNN model for 32 times to evaluate the stability of hyperparameters. Logistic regression models with the left atrial dimension, emptying fraction, and peak systolic GS as predictor variables were used for comparisons. Images from the apical 2-chamber (2-C) and 4-chamber (4-C) views had distinct features, leading to different CNN performance between settings; of them, the “4-C GS+4-C GSR” setting provided the highest performance index values. All four predictor variables used for logistic regression modeling were significant; however, none of them, individually or in any combined form, could outperform the optimal CNN model.

**Conclusion:** The novel approach using deep CNNs for learning features of left atrial curved M-mode speckle-tracking images seems to be optimal for classifying outcome status after AF ablation.

## Introduction

Speckle-tracking echocardiography (STE) is an imaging modality for analyzing and tracking small segments of the myocardium to provide greater detail for assessing global and regional cardiac motion and function. Recently, STE has been applied for assessing left atrial (LA) function, and has been proven to be superior to LA size as a predictor of atrial fibrillation (AF) recurrence after radiofrequency catheter ablation (RFCA) (1–3). LA longitudinal global strain (GS) and GS rate (GSR) are usually determined based on the average of six segmental values per view. Inaba et al. reported that the mean peak systolic GSR was significantly lower in patients with persistent AF than in age-matched controls (4). In addition to reduced LA deformation, LA mechanical dispersion is also pronounced in AF patients, accessed by calculating the standard deviations of segmental GS and GSR values (5). Alternatively, the curved M-mode color images of GS and GSR provide detailed spatiotemporal information of LA deformation mechanics. However, using visual estimation to precisely differentiate these images in challenging.

Deep learning, a class of machine-learning algorithms using multiple layers to progressively extract higher level features from raw input, has become a powerful method of classifying several diseases (6). Through model training, convolutional neural network (CNN) can interpret and analyze various features within a dataset and use them to learn how to generate an output label. CNNs have proven successful in learning patterns in images to aid experts in image-based diagnosis and classification (7). In the present study, we (a) assessed whether supervised deep learning with CNNs can be used to analyze curved M-mode STE images in patients with AF who have undergone RFCA and (b) analyzed whether the predictions of a deep CNN model are better than those of conventional logistic regression models with the LA dimension (LAD), emptying fraction (LAEF), apical 2-chamber peak systolic GS (2-C GS), and 4-chamber peak systolic GS (4-C GS) as predictor parameters.

## Materials and Methods

### Study Population

In this study, we retrospectively evaluated 606 consecutive AF patients (462 paroxysmal AF) who had undergone RFCA for symptomatic AF refractory to antiarrhythmic drugs between July 2008 and July 2019 at our institution. We obtained the detailed medical history of all patients regarding AF and related cardiovascular and systemic conditions. On the basis of the outcomes of AF ablation, we divided patients into the following two groups: Group 1, no AF recurrence with no antiarrhythmic drugs (*n* = 443, 73.1%) and Group 2, including both recurrence of atrial tachyarrhythmia responsive to antiarrhythmic drugs (*n* = 93, 15.3%) or refractory to antiarrhythmic drugs (*n* = 70, 11.6%). The Institutional Review Board of Chang Gung Memorial Hospital approved the study protocol (IRB No. 202000829B0), and written informed consent was obtained from all patients.

### Electrophysiological Study and RFCA

AF ablation was performed using a three-dimensional electroanatomical mapping system (CARTO, Biosense Webster, Diamond Bar, CA, USA) as previously reported (8). Briefly, all patients underwent RFCA under endotracheal intubation and general anesthesia. A 3.5-mm open-tip irrigated catheter (NaviStar Thermo-Cool, Biosense Webster) was percutaneously introduced through the right femoral vein for mapping and ablation. Circumferential pulmonary vein antral isolation with confirmation of entrance block was verified in all patients. If AF persisted or left atrial flutter occurred after pulmonary vein isolation, additional LA linear ablation was performed at the operator's discretion. External cardioversion was performed to restore sinus rhythm if RFCA failed to convert AF. Non-pulmonary vein triggers that reinitiated AF were ablated as necessary.

### Echocardiography

Patients underwent transthoracic echocardiography within 24 h after ablation. All patients were in sinus rhythm during echocardiography. Two-dimensional (2-D) echocardiography was performed using a commercially available ultrasonography machine (Vivid 9, General Electric Medical Health, Waukesha, WI, USA) with a 2.5-MHz phased-array transducer. All echocardiographic measurements were obtained in accordance with the guidelines of the American Society of Echocardiography (9). The 2-D LA volume was measured from the apical 4-chamber (4-C) view. LAEF was determined as the difference between the maximum LA volume in ventricular systole and the minimum LA volume in ventricular diastole, divided by the maximum LA volume (10).

**Figure 1:**
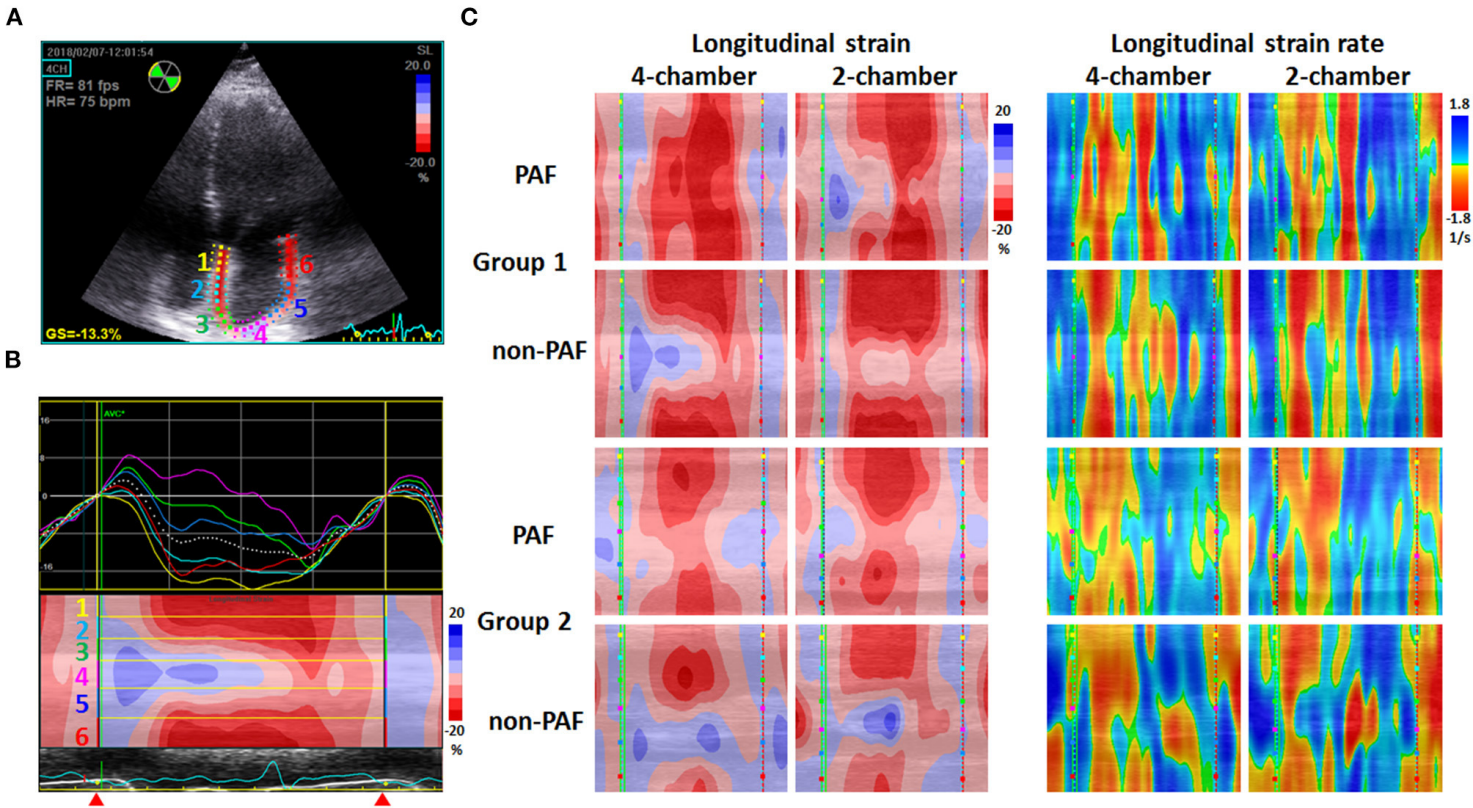
Examples of 2-D speckle-tracking echocardiography in two groups. **(A)** Echocardiograms of the apical 4-chamber view depicted the region of interest generated using speckle-tracking echocardiography software. The depicted left atrial wall was divided into six segments marked by different color. GS = −13.3% indicates peak systolic left atrial global strai*n* = −13.3%. **(B)** Above: the longitudinal strain curves of six segments with different color labeled in **(A)**; below: curved M-mode strain image representing the cyclic changes of strain at the region of interest along the time axis. Blue indicates positive values and red indicates negative values. The yellow lines separate the image into six portions labeled in **(A)**. Red triangles indicate the period of recording, which started at the end of the T wave (conduit phase), followed by the contraction phase. **(C)** Examples of curved M-mode strain (left) and strain rate (right) images obtained from apical 4-chamber and 2-chamber views in two groups, including paroxysmal atrial fibrillation (PAF) and non-PAF patients. Images in Group 1 show deeper red in the contraction phase and more homogeneous pattern of color distribution, indicating a better mechanical deformation and synchrony of the left atrium than those in Group 2.

STE images of the left atrium obtained in apical 4-C and 2-chamber (2-C) views with a frame rate between 60 and 100 frames/s were captured and stored digitally for offline analysis of LA GS and GSR (EchoPac PC, GE Vingmed, Horton, Norway). Special care was taken during echocardiographic image acquisition to ensure adequate LA tracking and avoid interference with the pulmonary veins and LA appendage to measure LA GS and GSR. The endocardium of the LA wall was manually traced starting from the medial/septal to the lateral mitral annulus in the apical 4-C view and inferior to anterior mitral annulus in the apical 2-C view, and was tracked by the 2-D speckle-tracking software along the border (Figure 1A). The operator manually adjusted segments that were not tracked. STE determined regional changes in length and was expressed as a positive value for lengthening or as a negative value for shortening. LA peak longitudinal systolic GS was assessed as the average of six segmental values per view.

The curved M-mode images of GS and GSR in both apical 4-C and 2-C views were also generated using the software, providing a unidimensional view of GS and GSR which illustrated the change in length and the change in strain/sec of the depicted LA wall along the time axis, respectively (Figures 1B,C). Curved M-mode STE images represented the cyclic changes of strain and strain rate at the region of interest along the time axis, starting from the end of the T wave (conduit phase) to the contraction phase and then the reservoir phase.

Intraclass correlation coefficients were calculated to quantify the intra-observer and inter-observer variability of GS in 48 randomly selected patients, measured first by the same investigator on two separate occasions for intra-observer variability, and then by two independent investigators for inter-observe variability. The two investigators were blinded to each other's measurements and the outcome status of AF ablation. Repeat measurement was made at the same cardiac cycle of the same image for each patient to avoid inherent variability caused by different cycle lengths.

### Follow-Up and Definition

Patients were followed up at 1 week, 1 month, 3 months, 6 months and every 3–6 months after RFCA and whenever required due to AF symptoms. Twelve-lead electrocardiograms and 24-h Holter ambulatory electrocardiograms were recorded after RFCA and when the patient experienced palpitation symptoms. Recurrence was defined as typical palpitation episodes for >30 s or atrial tachyarrhythmia on a 12-lead electrocardiogram, Holter monitoring, or pacemaker/implantable cardioverter-defibrillator interrogation records. Repeat RFCA as well as continuation of a previously ineffective antiarrhythmic drug were suggested to patients with AF recurrence.

### Processing of Data Import

**Figure 2:**
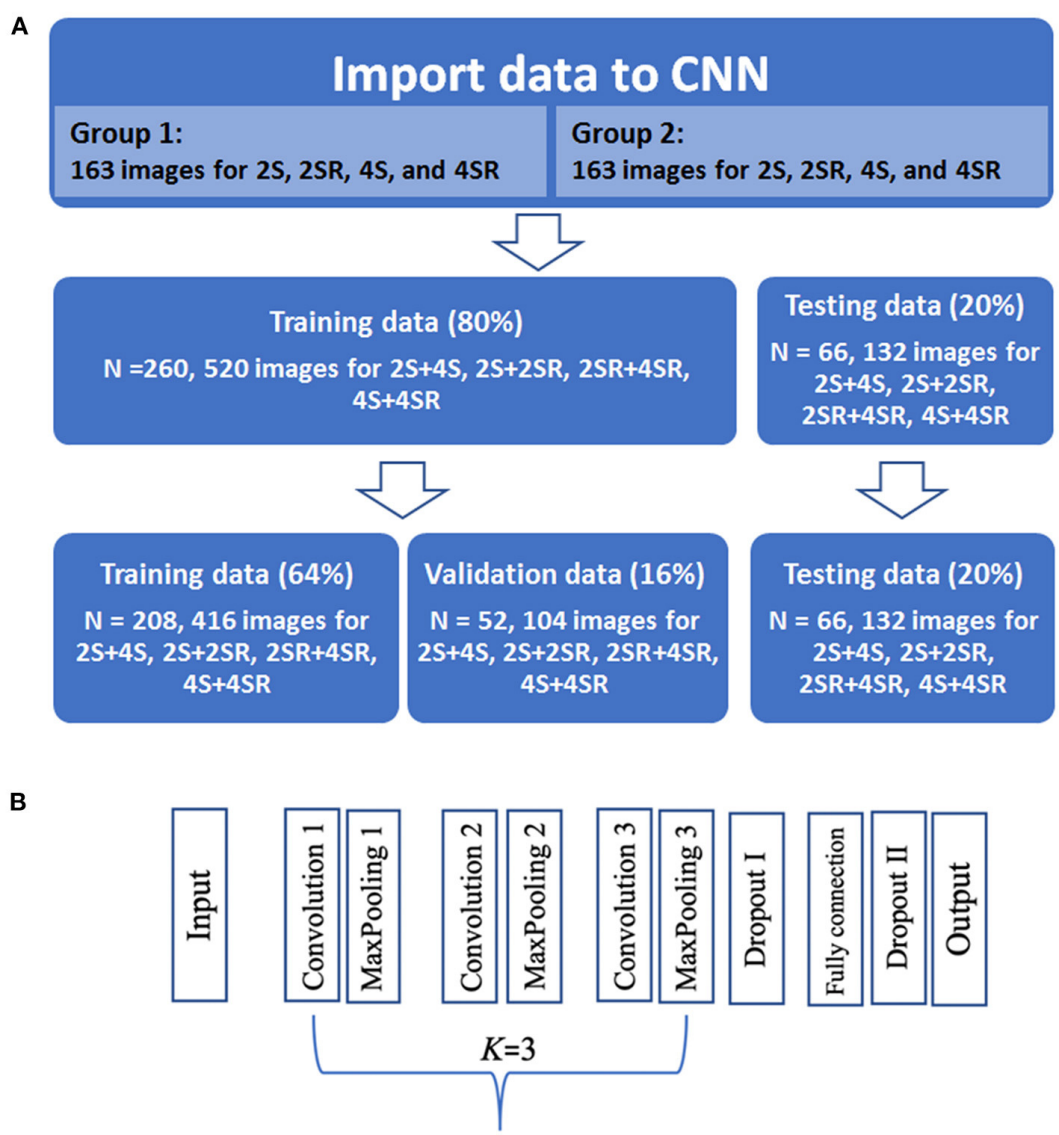
Import data and convolutional neural network (CNN) flow chart. **(A)** There was a total of 326 cases from which were split with 208 cases as the training set, 52 cases as the validation set, and 66 cases as the test set in building the CNN model. **(B)** Architecture of the final CNN model. 2S, apical 2-chamber strain map; 2SR, apical 2-chamber strain rate map; 4S, apical 4-chamber strain map; 4SR, apical 4-chamber strain rate map.

The curved M-mode RGB images of GS and GSR were extracted and standardized into portable network graphic images of 120 × 120 pixel. Because the classification ability was unclear, we used four combinations of the curved M-mode GS and GSR images: 2-C GS + 4-C GS (2S+4S), 2-C GSR + 4-C GSR (2SR+4SR), 4-C GS + 4-C GSR (4S+4SR), and 2-C GS + 2-C GSR (2S+2SR). Because of an imbalanced feature of the study sample, images from 163 randomly selected patients from Group 1 and 163 patients from Group 2 were used. In each replicate, 80% of the patient data were randomly selected as training data and the remaining were treated as testing data. The process of selecting the study samples is illustrated in Figure 2A.

### Architecture of the CNN

The CNN was then used to classify the subjects. As shown in Figure 2B, the CNN architecture was set to have an input layer, *K* sets of convolutional layer and max-pooling layer (feature maps), one flatten layer, *M* fully connected layers, and an output layer. For stable performance of the CNN model, the hyperparameters in the convolutional layer and *K* were determined dynamically (11). The 2-D convolutional layer built in Keras was used to construct the feature maps of the images (12). The hyperparameters in the convolutional layer included filter size, kernel size, stride, and padding. These parameters were determined dynamically among the parameter settings given in Table 1. We used the ReLu activation function for the CNN model. To determine the stability of the hyperparameters, the CNN model was performed repeatedly for 32 times. The decision on the final setting for the hyperparameters was based on the variability of the accuracy from 32 runs. The optimizer used the Adam algorithm with a learning rate set to 0.001 (13). CNNs were implemented using TensorFlow version 2.0 (Google Brain, 273 Mountain View, CA, USA) and Keras version 2.2.4 software (GitHub, San Francisco, CA) using Python version 3.5 programming language (Python Software Foundation, Beaverton, OR).

**Table 1:** Clinical and echocardiographic data of the study groups.

	**Group 1 (*n =* 443)**	**Group 2 (*n =* 163)**	***P*-value**
Age (year)	58 ± 11	61 ± 13	0.002
Gender (male, %)	317 (71.6%)	92 (56.4%)	0.001
BMI	25.3 ± 3.5	25.6 ± 3.8	0.360
Paroxysmal (%)	384 (86.7%)	78 (47.9%)	<0.001
AFD (year)	3.2 ± 3.0	4.3 ± 3.7	<0.001
Hypertension (%)	212 (47.9%)	88 (54.0%)	0.200
Diabetes mellitus (%)	73 (16.5%)	28(17.2%)	0.902
Dyslipidemia (%)	125 (28.2%)	62 (38.0%)	0.023
CAD (%)	22 (5.0%)	14 (8.6%)	0.119
Stroke (%)	22 (5.0%)	26 (16.0%)	<0.001
ESRD (%)	17 (3.8%)	14 (8.6%)	0.023
RHD (%)	3 (0.7%)	16 (9.8%)	<0.001
SSS (%)	41 (9.3%)	30 (18.4%)	<0.001
RFCA times	1.3 ± 0.6	1.6 ± 0.8	<0.001
**Echocardiographic data**			
LAD (mm)	40.34 ± 5.69	46.46 ± 7.39	<0.001
LAEF (%)	55.03 ± 9.99	38.78 ± 13.82	<0.001
LVEF (%)	67.22 ± 6.04	−13.13 ± 6.84	<0.001
MR degree			<0.001
No	94 (21.2%)	12 (7.4%)	
Mild	273 (61.6%)	75 (46.0%)	
Mild to moderate	76 (17.2%)	76 (46.6%)	
2-C GS (%)	−19.34 ± 6.60	−13.13 ± 6.84	<0.001
4-C GS (%)	−20.79 ± 38.40	−12.50 ± 6.05	0.005

*AFD, atrial fibrillation duration; BMI, body-mass index; CAD, coronary artery disease; ESRD, end-stage renal disease; LAD, left atrial dimension; LAEF, left atrial emptying fraction; LVEF, left ventricular ejection fraction; MR, mitral regurgitation; RFCA, radiofrequency catheter ablation; RHD, rheumatic heart disease; SSS, sick sinus syndrome; 2-C GS, apical 2-chamber peak systolic global strain; 4-C GS, apical 4-chamber peak systolic global strain*.

### Statistical Methods

Numbers and percentages were used to summarize the basic characteristics of the study sample. Two independent-sample *t*-tests were conducted to evaluate the association between continuous covariates and groups. A chi-square test was performed to assess the association between discrete covariates and groups. Logistic regression models were used to access the predictive power for group classification. The predictor variables included LAD, LAEF, 2-C GS, and 4-C GS. To understand the predictive power of these variables, seven settings were considered, including four individual predictors, LAEF+LAD, 2-C GS+4-C GS, and all four variables. Logistic regression models were constructed using the same selected subjects for training and testing the CNN model. The model was performed using SAS (Version 9.4, SAS Institute Inc., Cary, NC, USA).

The diagnostic performance of the CNN and the logistic regression models were evaluated using the confusion matrix and the area under the receiver operating characteristic curve (AUC) (14). The confusion matrix was constructed using a cutoff value of 0.5 and used to compute sensitivity, specificity, and accuracy. For the CNN model, the box chart was used to display the sampling distribution of the performance indices for 32 runs. Furthermore, because of the relatively small sample used, the final performance of the CNN model was determined by combining the results from 32 runs, which was also used to illustrate the ability of the source of images to predict abnormal status. The index values were computed three ways. For each subject, an abnormal status for one of the sources (i.e., “4S” or “4SR”) was defined as when the average probability of an abnormal status for 32 runs exceeded 0.5; for two subjects, an abnormal status for two sources (i.e., “4S+4SR”) was defined as when the average probability of an abnormal status for 64 runs exceeded 0.5.

## Results

Table 1 summarizes patients' baseline clinical characteristics. Group 1 patients were significantly younger than Group 2 patients. The proportions of men and paroxysmal AF were higher in Group 1. Group 1 also exhibited lower percentages of dyslipidemia, stroke, end-stage renal disease, rheumatic heart disease, and sick sinus syndrome; shorter AF duration; and fewer RFCAs. The echocardiographic data showed all four predictor variables used for logistic regression modeling were significant. Compared with Group 2, Group 1 demonstrated significantly smaller LAD, better LAEF, and more negative values of 2-C GS and 4-C GS.

There was excellent reproducibility of GS analysis. For intra-observer variability, the mean difference and intraclass correlation coefficient were 0.88 and 98.5%, respectively, for 4-C GS and 0.81 and 98.8%, respectively, for 2-C GS. For inter-observer variability, the mean difference and intraclass correlation coefficient were 1.30 and 97.0%, respectively, for 4-C GS and 0.99 and 98.6%, respectively, for 2-C GS.

### Logistic Regression Models

Table 2 lists the model estimates of the logistic regression models. When only one predictor variable was included, all four variables were significantly associated with outcome status after AF ablation (Table 2A). Table 2B presents the estimates of the logistic regression models when more than one predictor variable was included. Both LAD and LAEF were significant when they coexisted in the model. When 2-C GS and 4-C GS were included, 2-C GS became less important. When all four predictor variables were controlled for, 2-C GS and 4-C GS became non-significant, whereas LAD was only significant at *P* = 0.046. Overall, LAEF was the most influential variable for predicting outcome status after AF ablation using logistic regression.

**Table 2A:** Estimates for the logistic regression models with only one predicting variable.

	**LAD**			**LAEF**			**2-C GS**			**4-C GS**		
**Variables**	**Estimate**	**SE**	***P***	**Estimate**	**SE**	***P***	**Estimate**	**SE**	***P***	**Estimate**	**SE**	***P***
Intercept	−6.07	0.99	<0.001	4.49	0.63	<0.001	2.34	0.39	<0.001	2.79	0.43	<0.001
LAD	0.14	0.02	<0.001									
LAEF				−0.10	0.01	<0.001						
2-C GS							0.14	0.02	<0.001			
4-C GS										0.17	0.03	<0.001

**Table 2B:** Estimates for the logistic regression models with more than one predicting variables.

	**LAEF** **+** **LAD**			**2-C GS** **+** **4-C GS**			**LAEF** **+** **LAD** **+** **2-C GS** **+** **4-C GS**		
**Variable**	**Estimate**	**SE**	***P***	**Estimate**	**SE**	***P***	**Estimate**	**SE**	***P***
Intercept	0.43	1.50	0.774	2.98	0.44	<0.001	1.76	1.61	0.273
LAD	0.07	0.03	0.004				0.05	0.03	0.046
LAEF	−0.08	0.01	<0.001				−0.06	0.02	<0.001
2-C GS				0.05	0.03	0.076	0.01	0.03	0.700
4-C GS				0.13	0.03	<0.001	0.07	0.04	0.083

*LAD, left atrial dimension; LAEF, left atrial emptying fraction; SE, standard error; 2-C GS, apical 2-chamber peak systolic global strain; 4-C GS, apical 4-chamber peak systolic global strain*.

### Performance Indices of CNN Models on Assessing Curved M-Mode STE Images

**Figure 3:**
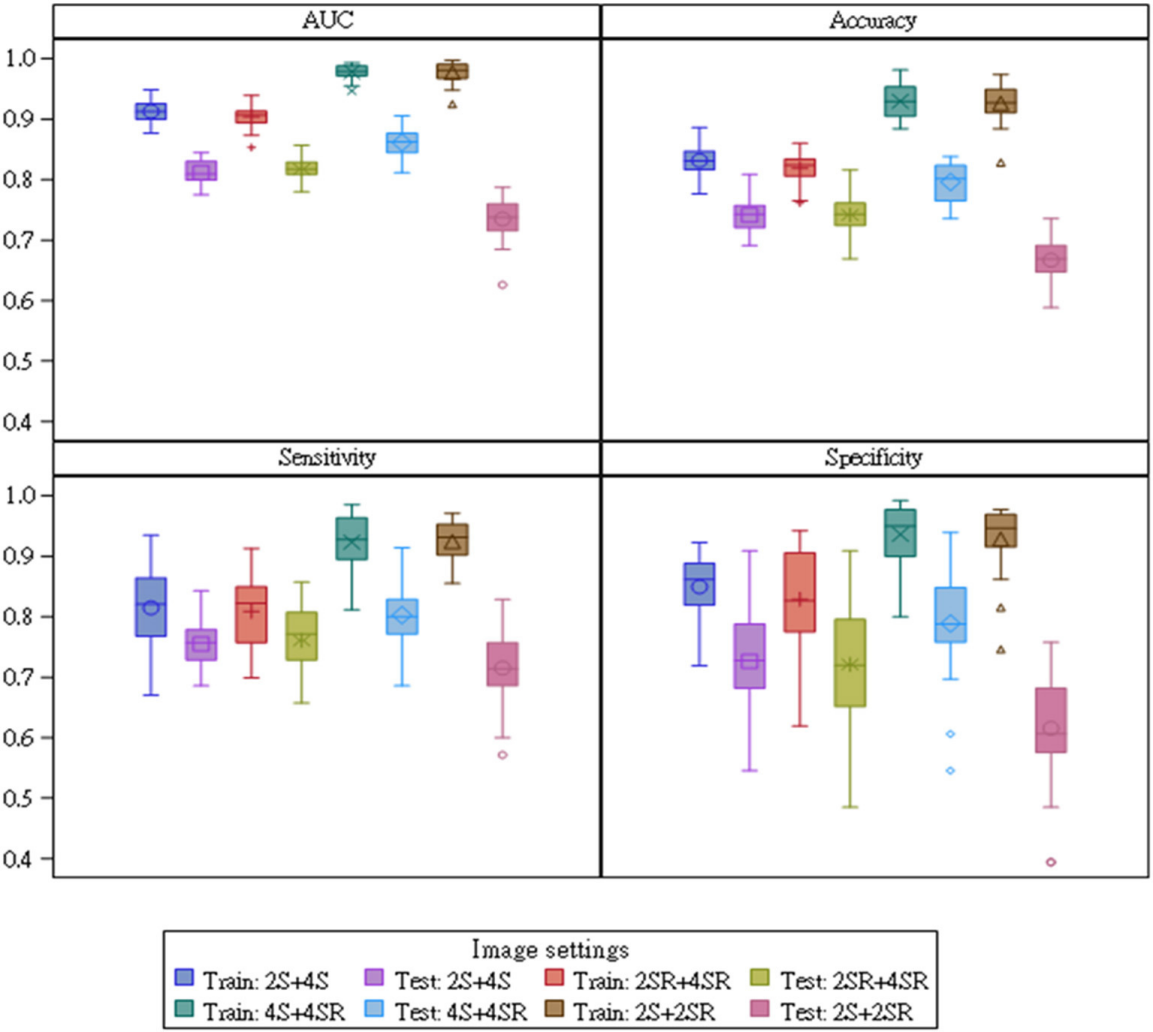
Comparisons of performance index values for 32 runs for four parameter settings. The 4S+4SR setting yielded the largest area under the receiver operating characteristic curve, accuracy, sensitivity, and specificity scores during training and testing. 2S, apical 2-chamber strain map; 2SR, apical 2-chamber strain rate map; 4S, apical 4-chamber strain map; 4SR, apical 4-chamber strain rate map.

The final settings for the hyperparameters are given in the last column of Table 3. The performance of classification algorithms was evaluated by computing the AUC, accuracy, specificity, and sensitivity. Figure 3 presents the box plot for the index values of 32 runs for the training and testing samples for four image settings, and the summarized results of statistics for all the performance indices were shown in Supplementary Table 1. Under the selected hyperparameters, the AUC derived from the training sample and testing sample was more than 0.8 for 2S+4S, 2SR+4SR, and 4S+4SR (the AUC derived from the testing sample for 2S+2SR was <0.8). The accuracy for 4S+4SR was more than 0.8, whereas that for 2S+4S and 2SR+4SR was lower than 0.8. The sensitivity and specificity derived from 4S+4SR were also higher than those for the other image settings. Overall, 4S+4SR had the best performance, whereas that of 2S+2SR was low when the testing sample was used.

**Table 3:** Parameter ranges for the hyperparameters in the convolutional layers.

**Layer**	**Hyper-parameters**	**Ranges for parameter**	**Final settings**
Convolution	Filter	16, 32, 64, 80, 95, 100, 105, 110, 128, 150, 256	32
	Kernel size	2, 3, 4, 5, 8, 10, 12	3
	Stride	1, 2, 3, 4, 5	2
	Dropout rate	0.25, 0.35, 0.4, 0.5	0.5
	*K*	1, 2, 3, 4	3
Max-polling	Kernel	2 (default)	2
	Stride	2 (default)	2
	Dropout rate	0.25, 0.3, 0.4, 0.5	0.5
Fully connection	Neuron (m)	128, 256, 512, 1,024	512
	*M*	1, 2	1

Table 4 presents the performance index values constructed by combining the results obtained from 32 runs. For each type, the performance indices were constructed based on three sources of data. For example, for 2S+4S, the performance index values were computed using results obtained individually from 2S and 4S and then computed with the 2S+4S results. For the 2S+4S setting, the performance index values computed from the results obtained using 4S images were much higher than those using 2S images for the testing sample. Moreover, the accuracy and specificity computed from the results obtained using 4S images were slightly higher than those using 2S+4S for the testing sample. Similarly, for the 2SR+4SR setting, the performance index values computed from the results obtained using 4SR were much higher than those using 2SR for the testing sample. That is, distinct features were observed for images from apical 2-C and 4-C views. Furthermore, the performance index values computed from 4S+4SR were much higher than that from 2S+2SR.

**Table 4:** Performance index values constructed from combining 32 runs.

		**Train**			**Test**		
**Image settings**	**Source**	**Accuracy**	**Sensitivity**	**Specificity**	**Accuracy**	**Sensitivity**	**Specificity**
2S+4S	2S	86.19	83.21	89.31	72.06	73.53	70.59
	4S	83.58	86.13	80.92	82.35	82.35	82.35
	2S+4S	88.81	89.05	88.55	80.88	85.29	76.47
2SR+4SR	2SR	83.21	81.75	84.73	72.06	76.47	67.65
	4SR	82.84	84.67	80.92	79.41	82.35	76.47
	2SR+4SR	87.69	86.86	88.55	82.35	85.29	79.41
4S+4SR	4S	94.40	92.70	96.18	85.29	88.24	82.35
	4SR	94.40	92.70	96.18	85.29	88.24	82.35
	4S+4SR	94.40	92.70	96.18	85.29	88.24	82.35
2S+2SR	2S	95.90	94.89	96.95	69.12	70.59	67.65
	2SR	95.90	94.89	96.95	69.12	70.59	67.65
	2S+2SR	95.90	94.89	96.95	69.12	70.59	67.65

*2S, apical 2-chamber strain map; 2SR, apical 2-chamber strain rate map; 4S, apical 4-chamber strain map; 4SR, apical 4-chamber strain rate map*.

### Comparisons of Model Performance

Table 5 provides the performance index values for all models. The CNN model using 4S+4SR images had the best performance, surpassing the logistic regression model using four predicting variables in terms of accuracy and sensitivity. The third best performance was achieved by the CNN model when using 2S+4S or 2SR+4SR images. The logistic regression model based on LAEF had similar sensitivity and specificity, whereas the one based on 2-C GS+4-C GS had similarly higher sensitivity but lower specificity.

**Table 5:** Performance indices for all models.

	**Train**			**Test**		
**Model settings**	**Accuracy**	**Sensitivity**	**Specificity**	**Accuracy**	**Sensitivity**	**Specificity**
C1: 2S+4S	88.81	89.05	88.55	80.88	85.29	76.47
C2: 2SR+4SR	87.69	86.86	88.55	82.35	85.29	79.41
C3: 4S+4SR	94.40	92.70	96.18	85.29	88.24	82.35
C4: 2S+2SR	95.90	94.89	96.95	69.12	70.59	67.65
M1: LAEF	76.12	74.45	77.86	79.41	79.41	79.41
M2: LAD	67.16	70.80	63.36	57.35	64.71	50.00
M3: 2-C GS	71.27	72.99	69.47	80.88	88.24	73.53
M4: 4-C GS	70.52	72.26	68.70	70.59	79.41	61.76
M5: LAEF+LAD	77.24	76.64	77.86	79.41	79.41	79.41
M6: 2-C GS+4-C GS	73.51	75.18	71.76	80.88	88.24	73.53
M7: LAEF+LAD+2-C GS+4-C GS	76.12	75.91	76.34	83.82	85.29	82.35

*C, CNN model; M, logistic model; 2S, apical 2-chamber strain image; 2SR, apical 2-chamber strain rate image; 4S, apical 4-chamber strain image; 4SR, apical 4-chamber strain rate image; LAD, left atrial dimension; LAEF, left atrial emptying fraction; 2-C GS, apical 2-chamber peak systolic global strain; 4-C GS, apical 4-chamber peak systolic global strain*.

## Discussion

The main finding of this study is that a deep CNN based on curved M-mode STE images (4S+4SR) achieved the highest prediction accuracy, sensitivity, and specificity compared with logistic regression models using LAEF, LAD, 2-C GS, or 4-C GS, individually or combined, as predictor parameters to assess outcome status after AF ablation.

### STE Imaging and CNN Models

Analysis of LA function is essential for the evaluation of patients with AF, and STE allows identifying those patients who are prone to develop AF and is a marker of LA fibrosis (15). Quantification of LA function using STE images enables evaluation of LA dysfunction due to AF (4), and is able to predict rhythm outcomes after AF ablation (16). A meta-analysis by Ma et al. indicated that LA STE images can facilitate the identification of patients with a high risk of AF recurrence in patients with paroxysmal AF, with a weighted mean AUC of 0.798 (17). STE is a sensitive tool to measure ultrastructural changes that affect LA mechanics before LA enlargement. LA deformation capacity measurement by STE provides a comprehensive assessment of atrial function and is more helpful for identifying abnormal atrial substrate than conventional echocardiographic variables, thus helping in the prediction of post-RFCA AF recurrence in both paroxysmal and persistent AF patients (18).

Furthermore, Sarvari et al. reported that inhomogeneous timing of LA contraction is potentially a predictor of AF recurrence after ablation (5). The segmental dysfunction corresponds to the LA substrate, and LA mechanical and electrical dysfunction coexist in the early phase prior to LA enlargement (19). STE can accurately assess regional myocardial function and timing. Chao et al. reported that applying artificial intelligence algorithms to the STE radial strain of the left ventricle can assist in identifying cardiac resynchronization therapy responders (20). They used complex mathematical methods to compute the difference and standard deviation of time to peak stain detected in each of the six regional strain curves and to convert two strain curves on two ventricular walls into a sequence of phase-space points (a total of 15 pairs) for phase space reconstruction. The authors applied a support vector machine using peak-strain timing and phase space reconstruction as parameters to build classifiers with an average accuracy, sensitivity, and specificity of >90%. For the left atrium, because the GSR curves include three peaks [strain rate during systole (SR-S), early diastole (SR-E), and atrial contraction (SR-AC)] to assess LA reservoir, conduit, and contractile function, it would be much complicated to apply artificial intelligence algorithms on the LA GS and GSR curves to build classifiers for patients post-AF ablation with different rhythm outcomes.

Alternatively, the spatiotemporal information of deformation can be displayed in curved M-mode RGB images of GS and GSR, on which red or blue, deep or light, and patterns of color distribution provide information of the direction, strength, and homogenization of LA deformation properties, respectively. These images can be useful for assessing LA function. However, these images have not been widely used clinically because visual assessment is inherently subjective and prone to classification error. Artificial intelligence in cardiac imaging is a fast-moving field (21), and deep learning is a form of machine learning devised to mimic the way the visual system works (22). Deep CNN models were reported to correctly classify all types of echocardiogram recordings (M-mode, Doppler, still images, and videos) (23). Our findings indicate that a deep CNN can successfully incorporate spatiotemporal features from these RGB images into an overall assessment of LA deformation mechanics. This deep CNN model may also be applicable to left ventricular studies for identifying cardiac resynchronization therapy responders in patients with severe heart failure and left bundle branch block.

Previously, we reported that LAEF provides the optimal prognostic information regarding the risk stratification of AF patients undergoing RFCA (10). In this study, we placed the P-wave in the middle of the time axis by setting zero reference at the end of the T-wave corresponding to the onset of mitral valve opening for a better illustration of LA shortening on curved M-mode images of GS. Because the LA wall is longest at this point, LA strain values were negative, and a prominent red color can be seen in the middle of the images. As shown in Figure 1C, Group 1 had a deeper and more homogeneous red color zone in the middle of the map than Group 2, indicating more effective and synchronized LA shortening. Current strain software packages usually provide an electrocardiogram trigger as a zero reference, which is frequently situated at the upslope of the R-wave as a surrogate for end-diastole or at the onset of LA contraction. Both are the currently used methods reported in the latest European standardization documents (24, 25). Because the entire strain curve changes its amplitude depending on the definition of zero reference, LA GS values obtained by setting zero reference at the peak of the LA strain curve in this study would be much smaller than those obtained by setting zero reference at the nadir of the LA strain curve in the currently used method.

### Logistic Regression Models and CNN Models

By incorporating the spatiotemporal information of LA deformation properties, this study demonstrated that the STE image-based CNN model (4S+4SR) had the best performance and surpassed even the logistic regression model using all four parameters. It implies that mechanical synchrony and empty fraction of the left atrium both are important predictors of post-RFCA AF recurrence, and also demonstrates the potential advantages of supervised deep learning with CNNs for image classification tasks. Why the apical 4-C view-acquired images provided better discriminating information than those acquired through the apical 2-C view for CNNs is unclear. A possible reason is that the apical 4-C view is the easiest and most reproducible to perform. To increase feasibility, the consensus of the European Association of Cardiovascular Imaging/American Society of Echocardiography/Industry Task Force to Standardize Deformation Imaging (24) recommends that using the LA longitudinal strain values obtained from a single non-foreshortened apical 4-C view is acceptable.

Tracing the LA outline manually is time consuming. Automated measurement of the left ventricular longitudinal strain is feasible (26). Using the TOMTEC automatic cardiac measurement software package and CNN models, the UCSF Echocardiography Laboratory reported that the automated global longitudinal strain values deviated from manual values by an absolute value of 1.4% (relative value of 7.5%) (27). Therefore, a future goal would be to achieve completely automatic generation and interpretation of curved M-mode speckle-tracking images of the left atrium to provide rapid and reproducible assessment of LA deformation properties.

This study has some limitations. A large sample size is required to achieve sufficient CNN model performance. Because our patient number was limited, the performance index of the deep CNN may have been underestimated in this study. Moreover, results in the test datasets were substantially worse than in the training, which suggests overfitting because of small training datasets. Even if we have repeated the algorithms 32 times and summarized the result in terms of 32 runs to reduce the random selection bias in the CNN modeling, increasing the size of training dataset with growing number of AF patients who have undergone RFCA is necessary to validate and strengthen the performance of the proposed CNN model. Finally, all results were derived from retrospective data. A prospective validation study is required to verify the reliability of the proposed CNN model before this approach is considered for clinical use.

## Data Availability Statement

The raw data supporting the conclusions of this article will be made available by the authors, without undue reservation.

## Ethics Statement

The studies involving human participants were reviewed and approved by The Institutional Review Board of Chang Gung Memorial Hospital (IRB No. 202000829B0). The patients/participants provided their written informed consent to participate in this study.

## Author Contributions

Y-TH designed and performed CNN, logistic regression modelling, and interpret data. H-LL analyzed strain and strain rate data, including off-line acquisition of cured M-mode images, and wrote the draft of the manuscript. P-CC, C-HL, H-TW, and H-TL collaborated in electrophysiological procedures, echocardiography, and data analyses. All of them contributed significantly to the execution of this study. M-SW is the senior investigator who helped design the study and participated in electrophysiological studies and ablation. F-CL is the senior investigator who helped with speckle-tracking data analysis. C-CC is the corresponding author who designed the study, interpreted the data, and revised the manuscript critically. All authors contributed to the article and approved the submitted version.

## Conflict of Interest

The authors declare that the research was conducted in the absence of any commercial or financial relationships that could be construed as a potential conflict of interest.
