# Supplementary material for: A Novel Approach for Predicting Atrial Fibrillation Recurrence After Ablation Using Deep Convolutional Neural Networks by Assessing Left Atrial Curved M-Mode Speckle-Tracking Images
Source: Front Cardiovasc Med. 2021 Jan 22;7:605642. doi: 10.3389/fcvm.2020.605642 (PMC7862331; doi:10.3389/fcvm.2020.605642)
Supplement: Supplementary file 1 [file Table_1.docx]

**Supplementary Table 1** Summary of mean values and standard errors of performance indices for 32 runs.

|  | Train | | | | | | | | Test | | | | | | | |
| --- | --- | --- | --- | --- | --- | --- | --- | --- | --- | --- | --- | --- | --- | --- | --- | --- |
| Image settings | AUC | | Accuracy | | Sensitivity | | Specificity | | AUC | | Accuracy | | Sensitivity | | Specificity | |
|  | Mean | SE | Mean | SE | Mean | SE | Mean | SE | Mean | SE | Mean | SE | Mean | SE | Mean | SE |
| 2S+4S | 0.913 | 0.016 | 0.832 | 0.023 | 0.815 | 0.061 | 0.850 | 0.053 | 0.812 | 0.020 | 0.741 | 0.029 | 0.755 | 0.041 | 0.726 | 0.086 |
| 2SR+4SR | 0.904 | 0.018 | 0.819 | 0.023 | 0.809 | 0.059 | 0.829 | 0.082 | 0.817 | 0.018 | 0.741 | 0.031 | 0.761 | 0.054 | 0.721 | 0.099 |
| 4S+4SR | 0.978 | 0.012 | 0.930 | 0.030 | 0.923 | 0.044 | 0.936 | 0.050 | 0.861 | 0.023 | 0.796 | 0.031 | 0.803 | 0.051 | 0.789 | 0.083 |
| 2S+2SR | 0.977 | 0.016 | 0.926 | 0.031 | 0.924 | 0.036 | 0.929 | 0.053 | 0.735 | 0.034 | 0.667 | 0.034 | 0.715 | 0.064 | 0.616 | 0.086 |

AUC, area under the receiver operating characteristic curve; SE, standard error; 2S, apical 2-chamber strain map; 2SR, apical 2-chamber strain rate map; 4S, apical 4-chamber strain map; 4SR, apical 4-chamber strain rate map
